# The Effect of Remelting on the Physical Properties of Borotellurite Glass Doped with Manganese

**DOI:** 10.3390/ijms14011022

**Published:** 2013-01-07

**Authors:** Syed Putra Hashim Syed Hashim, Haji Abdul Aziz Sidek, Mohamed Kamari Halimah, Khamirul Amin Matori, Wan Mohamad Daud Wan Yusof, Mohd Hafiz Mohd Zaid

**Affiliations:** Glass and Ultrasonics Studies Centre (GUSC), Department of Physics, Faculty of Science, Universiti Putra Malaysia, 43400 UPM Serdang, Selangor, Malaysia; E-Mails: hyzzam2003@yahoo.com (S.P.H.S.H.); halimah@science.upm.edu.my (M.K.H.); khamirul@science.upm.edu.my (K.A.M.); wmdaud@science.upm.edu.my (W.M.D.W.Y.); mhmzaid@gmail.com (M.H.M.Z.)

**Keywords:** borotellurite glass, density, bridging oxygen, FTIR spectra

## Abstract

A systematic set of borotellurite glasses doped with manganese (1–*x*) [(B_2_O_3_)_0.3_(TeO_2_)_0.7_]-*x*MnO, with *x* = 0.1, 0.2, 0.3 and 0.4 mol%, were successfully synthesized by using a conventional melt and quench-casting technique. In this study, the remelting effect of the glass samples on their microstructure was investigated through density measurement and FT-IR spectra and evaluated by XRD techniques. Initial experimental results from XRD evaluation show that there are two distinct phases of glassy and crystallite microstructure due to the existence of peaks in the sample. The different physical behaviors of the studied glasses were closely related to the concentration of manganese in each phase. FTIR spectra revealed that the addition of manganese oxide contributes the transformation of TeO_4_ trigonal bipyramids with bridging oxygen (BO) to TeO_3_ trigonal pyramids with non-bridging oxygen (NBO).

## 1. Introduction

Tellurite glass is an extremely promising material for laser and nonlinear applications in optics due to some of its essential characteristic features, such as high density, high refractive index, low phonon maxima, low melting temperature and excellent transparency in the far infrared region [1,2]. Furthermore, tellurite glass has a low melting point and is nonhygroscopic, which is an advantage when compared to borate and phosphate glasses. These types of glasses are extremely stable against devitrification, nontoxic and resistant to moisture for long periods of time [2]. It is widely recognized that the refractive index, *n*, and density, ρ, of many common glasses can be varied by changing the base glass composition [3]. In binary tellurite glasses, the basic structural unit of TeO_4_ is trigonal bipyramid (tbp) with a lone pair of electrons, and the structural units permit Te–O–Te bonding for glass formation [4].

The addition of tellurite to any other glass former or network modifier, such as B_2_O_3_, is of scientific and practical interest and may lead to the formation of interesting structural units that affect the physical properties of the glass network [5]. As reported earlier, the boron coordination number in the borate glass changed from three to four as more alkaline content was added into the system where the network linkage was increased. In contrast, the Te coordination number changed from four to three by the cleavage of the tellurite glassy matrix [6,7]. In fact, the presence of TeO_2_ in the matrix of alkali borate glasses decreases its hygroscopic nature; however, it improves the quality and enhances the IR transmission [8,9]. The role of alkali, alkaline earth, and transition metal oxides (TMO) in the borotellurite network is to modify the host structure through the conversion of the structural units of the borate system from [BO_3_] to [BO_4_] and the tellurite network from trigonal bipyramid [TeO_4_] to trigonal pyramid [TeO_3_] [10–13]. The elastic moduli of borotellurite glasses (TeO_2_–B_2_O_3_) have been reported and discussed based on the bond compression model [10,12].

In this work, borotellurite glasses doped with manganese oxide (MnO) in the form of (1–*x*) [(B_2_O_3_)_0.3_(TeO_2_)_0.7_]-*x*MnO, with *x* = 0.001, 0.002, 0.003 and 0.004, were prepared by using a conventional melt and quench-casting technique. The main objective of this work is to determine the optimum concentration of manganese needed to prepare the glass by examining amorphous characteristics using X-ray diffraction. The effect of remelting each glass sample is also studied.

## 2. Results and Discussion

**Figure 1:**
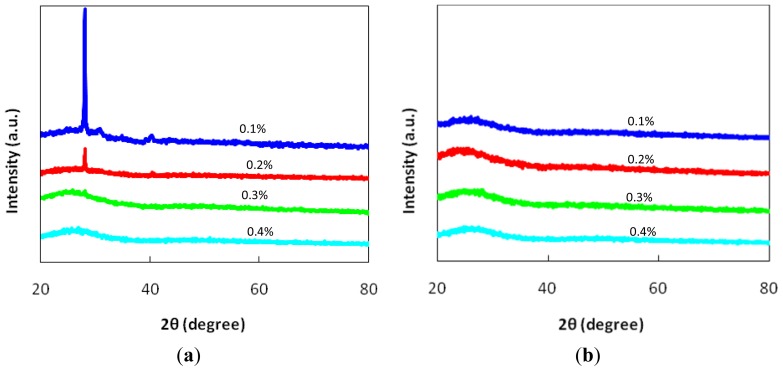
The XRD pattern of (**a**) the original sample and (**b**) the remelted MnO–B_2_O_3_–TeO_2_ glass samples.

The XRD patterns for the various compositions of (1–*x*)[(B_2_O_3_)_0.3_(TeO_2_)_0.7_]-*x*MnO glasses are shown in Figure 1, and the existence of a peak for MnO concentrations of 0.1 mol% and 0.2 mol%, which is related to the existence of a crystalline phase in the samples, is shown in Figure 1a. The addition of the MnO has disturbed the borotellurite glass system. In general, crystal growth can occur at any temperature if a seed crystal is available. It may establish and enhance crystal growth inside a system where a detectable growth rate can occur at any temperature below the *T*_m_. These crystalline peaks correspond to cubic manganese telluride borate, Mn_3_B_7_O_12.65_Te_0.85_, with the reference number 00-026-1255 [11–13]. At 0.3 mol% and 0.4 mol% MnO, the glass systems are in the amorphous state due to the optimum concentration of MnO, where it acts as a stabilizer for the glass system. Figure 1a also shows that no sharp peaks exist at 0.3 mol% and 0.4 mol% of MnO [10–15].

Manganese ions seem to exist in the Mn^2+^ and Mn^3+^ states in the glass network. However, at lower concentration of MnO, a majority of the manganese ions are in the Mn^2+^ state. The linkage of the Mn^2+^ and Te^4+^ ions is expected to be extremely weak because the difference in ionic radii of the Mn^2+^ (0.8 Å) and Te^4+^ (0.84 Å) is high when compared to that of Mn^3+^ (0.58 Å) and Te^3+^ (0.52 Å) ions [15].

To study the remelting effect on the glass structure, all of the glass samples were then remelted. In general, the features of the XRD patterns confirm that all of the remelted glasses are in the amorphous state (Figure 1b), as indicated by the broad hump that occurs at approximately 2θ = 20°–30° for all of the remelted glass samples.

**Figure 2:**
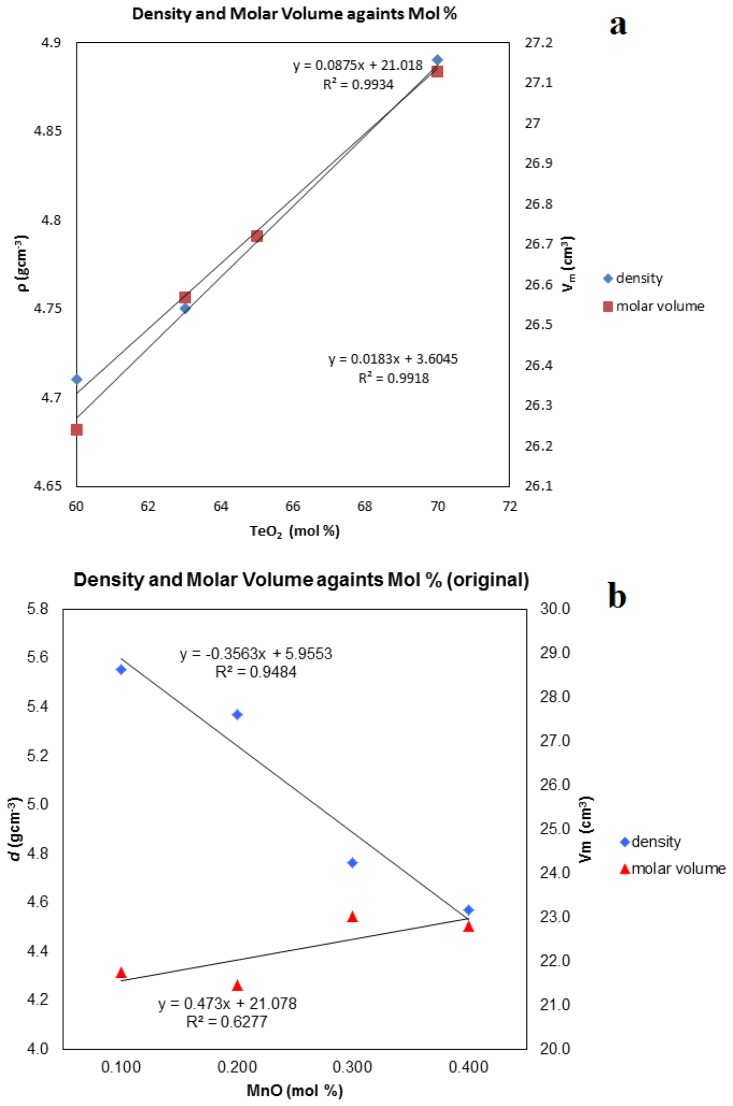

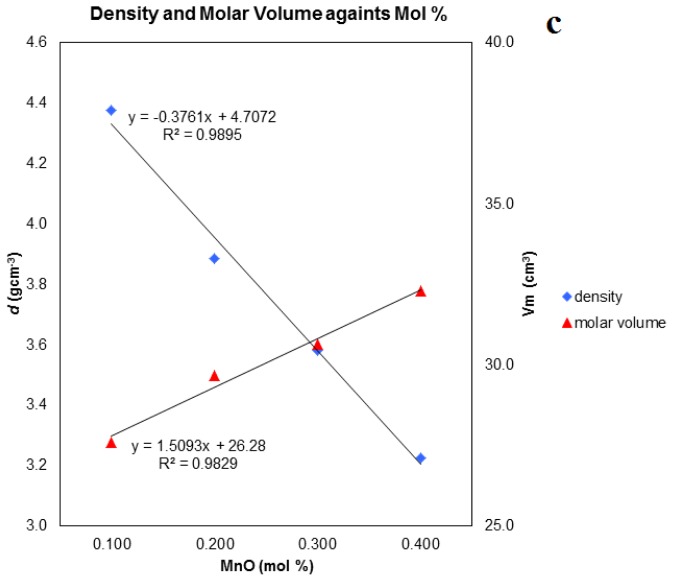
The density and molar volume of (**a**) borotellurite glasses; (**b**) the original (100–*x*) [(B_2_O_3_)_30_(TeO_2_)_70_]-*x*MnO glass samples and (**c**) the remelted samples.

All of the prepared glasses are free of bubbles, purple in color and of good quality. The density (ρ) and molar volume (*V*_m_) of the glass samples are shown in Figure 2 and Table 1. The density of the pure borotellurite increased steadily with the addition of TeO_2_ into the glass structure, as depicted in Figure 2a. It can be observed that the density decreases gradually with the compositions for both glasses before and after remelt with an addition of MnO (see Figure 2b,c).

The density results as depicted in Table 1 show that as the manganese cation concentration increases, the glass structure becomes more open, allowing for the likely creation of more nonbridging oxygen (NBO) [15,16]. Additionally, Figure 2 shows that the molar volume increases with the introduction of manganese content. In the present samples, the glass densities vary from 4.57 to 5.56 gcm^−3^ and 3.23 to 4.38 gcm^−3^ after remelt, revealing a rather linear relationship with the manganese content. However, there are slight differences in the density between the before and after remelt (Figure 2c) samples due to the existence of a crystalline phase inside the glass system. The occurrence of crystal growth causes a decrease of NBO. The remelting effect of this glass network is the reconstruction of the structure of the glass system and an increase of NBO, causing a decrease in the density.

The composition dependence of the molar volume gives information about the coordination state of the manganese cations. The density and molar volume for these glasses are compatible with the ionic size, atomic weight, and amount of different elements in the glasses.

**Figure 3:**
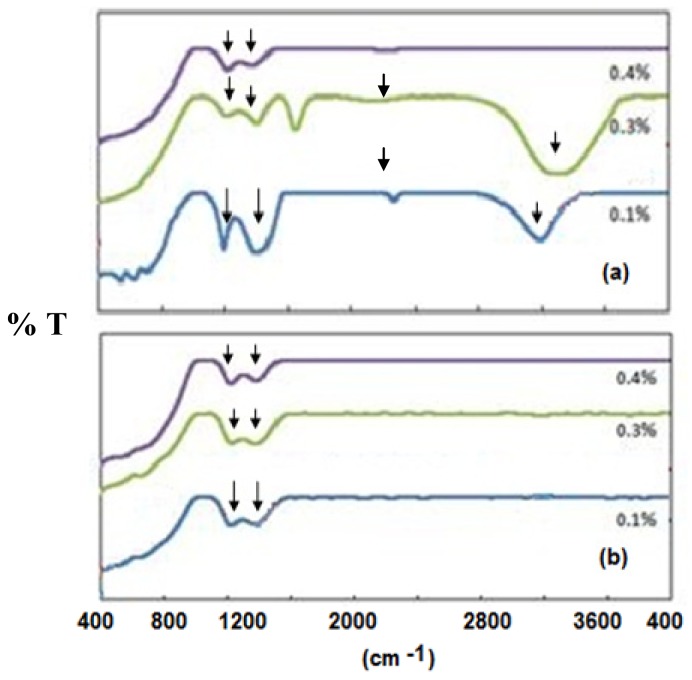
Selected FT-IR spectra of (**a**) the original sample; (**b**) the remelted sample.

The experimental FTIR spectra for the borotellurite glasses doped with manganese (100–*x*) [(B_2_O_3_)_30_(TeO_2_)_70_]-*x*MnO, with *x* = 0.1, 0.2, 0.3 and 0.4 mol%, are presented in Figure 3a,b. The FTIR spectral bands of the glasses and their assignments are summarized in Table 2. The data were analyzed following the method proposed by Condrate [17], comparing the experimental data of the glasses with those of their corresponding crystalline compounds.

The present study shows that the quantitative evolution of these glass structures are greatly influenced by the MnO concentration. The addition of MnO to the glass matrix leads to a drastic reduction in intensity between the ~520 and ~650 cm^−1^ absorption bands due to the Te–O bond between the trigonal bypiramidal unit [TeO_4_] and bridging oxygen and also contributes to the specific vibration of the Mn–O bond [18–20]. If we take into account the Mn–O bond vibrations’ contribution to the ~520 cm^−1^ absorption band, it seems that the controlled addition of manganese ions constricts, to a large degree, the bending motion of different boron-oxygen bonds and gradually increases the number of Mn–O linkages.

The vibration of the B-O arrangement in the infrared region of 400–1400 cm^−1^ is more profound [20,21]. The medium absorption observed at ~1200 cm^−1^ is attributed to the B–O asymmetric stretching of the tetrahedral BO_4_ [18] and orthoborate group [21,22]. The intensity of this band decreases for the original samples from *x* = 0.1% to *x* = 0.4%. As for the remelted sample, the intensities of these bands remain the same as the concentration of manganese ions is increased.

The band intensity at ~1400 cm^−1^ is due to the asymmetric stretching of the B–O bond from the [BO_3_] trigonal unit in varied borate rings [18,22,23]. The band intensity decreases with the increase in concentration of manganese ions for the original samples.

The band intensities between ~1600 cm^−1^ and ~3200 cm^−1^ are assigned to the bending of O–H and the asymmetric stretching of O–H, respectively [24,25]. The occurrence of the O–H bond inside the glass for the *x* = 0.1% to *x* = 0.3% original samples corresponds to the existence of a crystal structure of manganese telluride borate, which is highly soluble and simply reacts with H_2_O. As the band intensity at ~1200 cm^−1^ and ~1400 cm^−1^ decreases, the band intensity at ~1600 cm^−1^ and ~3200 cm^−1^ also decreases and eventually disappears for the *x* = 0.4% original sample. There is no bending of O–H or stretching of O–H for the remelted sample because no crystal structure exists in the glass matrix, as affirmed by Figure 1b.

## 3. Experimental Section

A 13 g batch of the (1–*x*) [(B_2_O_3_)_0.3_(TeO_2_)_0.7_]-*x*MnO glass system, with *x* = 0.1, 0.2, 0.3 and 0.4 mol%, was prepared by mixing all of the components together. The mixture was mechanically ground and homogenized using an agate mortar for 15 min. The mixture was then preheated inside an alumina crucible in an electrical furnace for half an hour at a temperature of 400 °C. The preheated mixture was then transferred to the second furnace for one hour at a temperature of 950 °C. To improve homogeneity, the crucible was constantly shaken inside the furnace. The melt was then poured into a stainless steel cylindrically shaped split mold, which was preheated at 350 °C before being transferred to an annealing furnace for two hours at 350 °C. After two hours, the furnace was allowed to cool to room temperature.

The cylindrically shaped samples obtained were then cut using low speed diamond blade to make parallel fine surfaces of 6 mm thickness. The unused part of the glass was taken and ground in to a fine powder. The fine powders were then remelted, and the entire procedure above was then repeated to examine the remelting effect. The entire procedure for the remelted sample preparation was the same, including the preheating, melting and annealing temperature, so that the conditions of the samples could be maintained as the conditions of the original samples.

The amorphous nature of the glasses was ascertained from XRD analysis using an X-ray Diffractometer (PAnalytical (Philips) X’Pert Pro PW 3040/60).

The density of each glass was measured using the Archimedes method with distilled water as the immersion liquid. A bulk glass was weighed in air (*W*_air_), immersed in distilled water and then reweighed (*W*_dw_), where the density of the distilled water was 1.00 g cm^−3^. The relative density is given as ρ_s_ = ρ_dw_ (*W*_air_/*W*_dw_) [26].

## 4. Conclusions

Borotellurite glass doped with manganese oxide was prepared by the melt-quenching technique. The XRD, density and molar volume of the glasses were discussed. The overall features of the XRD curves showed that the occurrence of peaks for the 0.1 mol% and 0.2 mol% samples are due to the existence of crystal seeds inside this glass structure. However, for the 0.3 mol% sample, the XRD pattern confirms the amorphous nature of the glass. The remelting effect, on the other hand, reconstructs the glass structure and avoids the nucleation of crystal growth. This result was confirmed by the XRD patterns showing the amorphous nature of all of the remelted glasses. Density was observed to decrease with the increase of MnO in the glass and the remelting effect. However, a slightly different density was shown for both glass systems due to the existence of crystallites inside the remelted glass network. This effect reconstructed the structure of the glass system and increased the amount of nonbridging oxygen inside the system, causing the density to decrease after it was remelted, which was proven by the FT-IR spectral analysis.

## Figures and Tables

**Table 1:** The glass composition (mol%), density and molar volume of (100–*x*) [(B_2_O_3_)_30_(TeO_2_)_70_]-*x*MnO.

MnO	B_2_O_3_	TeO_2_	ρ (g/cm^3^)	ρ_remelt_ (g/cm^3^)	*V*_m_ (cm^3^)	*V*_m,remelt_ (cm^3^)
0.1	29.97	69.93	5.555	4.3773	22.734	27.597
0.2	29.94	69.86	5.370	3.885	23.507	29.668
0.3	29.91	69.79	4.764	3.5798	26.483	30.649
0.4	29.88	69.72	4.569	3.2253	27.604	32.300

**Table 2:** Frequencies and their assignments for the FT-IR spectra of (100–*x*) [(B_2_O_3_)_30_(TeO_2_)_70_]-*x*MnO.

Peak positions (cm^−1^)	Assignments
~520	Corresponds to the Mn–O bond
~650	The Te–O bond of the trigonal bypiramidal unit [TeO_4_] with NBO and the contribution of the specific vibration of the Mn–O bond
~1200	The asymmetric stretching vibration of the B–O bond for the tetrahedral and orthoborate group
~1400	The asymmetric stretching of the B–O bond from the [BO_3_] trigonal unit in diverse borate rings
~1600	The bending of O–H
~3200	The asymmetric stretching of O–H
